# Honey Adulteration Detection via Ultraviolet–Visible Spectral Investigation Coupled with Chemometric Analysis

**DOI:** 10.3390/foods13223630

**Published:** 2024-11-14

**Authors:** Elisabeta-Irina Geană, Raluca Isopescu, Corina-Teodora Ciucure, Cristiana Luminița Gîjiu, Ana Maria Joșceanu

**Affiliations:** 1 National R&D Institute for Cryogenics and Isotopic Technologies—ICSI Ramnicu Valcea, 4th Uzinei Street, 240050 Ramnicu Valcea, Romania; irina.geana@icsi.ro (E.-I.G.); corina.ciucure@icsi.ro (C.-T.C.); 2 Faculty of Chemical Engineering and Biotechnologies, National University of Science and Technology POLITEHNICA Bucharest, 011061 Bucharest, Romania; raluca.isopescu@upb.ro (R.I.); ana.josceanu@upb.ro (A.M.J.)

**Keywords:** honey adulteration, sugar syrups, UV–Vis spectra, machine learning instruments, PCA, LDA, PLS, neural networks

## Abstract

Any change in the composition or physicochemical parameters of honey outside the standardized intervals may be deemed fraud, irrespective of direct introduction of certain substances or feeding honeybees with syrups. Simple and rapid tools along with more sophisticated ones are required to monitor fraudulent practices in the honey trade. In this work, UV–Vis spectroscopy was used to identify and quantify six Romanian honey types (five monofloral and one polyfloral) mixed with commercially available corn syrup, corn syrup with plant extracts, inverted syrup, and fruit syrup at different concentrations (5%, 10%, 20%, 30%, 40%, and 50%). Relevant spectral features were used to develop a neural model, which was able to pinpoint adulteration, regardless of the honey and adulterant type. The proposed model was able to detect adulteration levels higher than 10%, thereby serving as a cost-effective and reliable tool to monitor honey quality.

## 1. Introduction

Honey and beehive products enjoy significant importance on the social market alongside other natural resources of essential amino acids, polyphenols, flavones, organic acids, vitamins, and enzymes, biologically active compounds that represent true biological weapons against various pathogens and ultraviolet radiation [1,2] and protect against diabetes, cancer, cardiovascular, pulmonary, liver, and neurodegenerative diseases [3,4]. With over 200 major and minor components, all contributing to its special anti-inflammatory, antioxidant, and sensorial qualities, and a constantly increasing market value, honey ranks among the three most adulterated foods, along with wine, milk, and olive oil [5,6,7].

Advances in sample analysis technology coupled with the availability of novel information technology (IT) instruments have expanded the applications of machine learning tools in various domains, including food quality control [8]. Numerous spectroscopic techniques, such as Raman, Fourier-transform infrared (FTIR), near-infrared (NIR), and UV–Vis, combined with chemometrics, have proven their efficiency in identifying honey adulteration [7]. Although FTIR and Raman spectroscopies are generally preferred due to their comprehensive data, UV–Vis spectroscopy offers advantages in terms of cost and rapidity. Recently, researchers have focused on combining UV–Vis spectral data with adequate machine learning tools to design reliable instruments for food quality control [9].

Honey adulteration is carried out directly, by deliberately adding certain substances into it, or indirectly, by feeding the honeybees with the adulterating compound [10]. Mixing inexpensive honeys, such as rape honey, with acacia honey [11], and treating bees with antibiotics for disease prevention or heavy metals contamination as a result of apicultural practices [12] also fall under the category of adulteration. Although many adulterating agents may not pose immediate health risks, any changes in the composition or physicochemical parameter values outside the standardized intervals may be considered a fraud and should be prohibited in the honey trade [13]. The adulteration with sugar syrups modifies the bioactive composition and implicitly alters the therapeutic properties of honey [14].

The international standards of honey quality defined in the Codex Alimentarius Standard [15] and the EU Directive 110/2001 [16] define several physicochemical parameters, leaving national standards to further detail the sugar content (total sugar, total reducing sugar, invert sugar, fructose, glucose, fructose/glucose ratio), acidity (pH, free acidity, lactonic or total acidity), nitrogenous compounds (protein content, nitrogen content, proline content, diastase index, invertase index), phenolic compounds, hydroxymethyl furfural, minerals, trace elements, water activity, viscosity, glass transition temperature, and color.

Typical adulteration is conducted by adding inverted sugar (IS) or syrups (corn syrup—CS; high-fructose corn syrup—HFCS; high-fructose inulin syrup—HFIS; inverted cane sugar syrup—ICSS; beet syrup—BS; rice syrup—RS; date syrup—DS; jaggary syrup—JS) as their illicit levels are difficult to detect by sugar analysis, due to their properties being similar to those of natural honey [11]. Stable carbon isotope ratio analysis (SCIRA) is the standard technique for direct and indirect adulteration detection, highlighting even 7% adulterants extracted from C4 plants [17]. High-performance liquid chromatography with isotope ratio mass spectrometric detection (HPLC-IRMS) lowers the detection limit (LOD) to 2% for C3 sugar adulterants [18]. The reliability and accuracy of chromatographic techniques are indisputable, as various adulterant markers have been successfully identified using gas chromatography (GC) [19], liquid chromatography (LC) [20], and high-performance anion-exchange chromatography with pulsed amperometric detection (HPAEC-PAD) [21]. The LOD further drops to 1% when reversed-phase solid-phase extraction coupled with HPAEC-PAD is used to quantify corn sugar syrup in honey [22]. Though powerful, all these techniques are resource-intensive in terms of time, consumables, personnel, and cost. Therefore, more affordable techniques have been proposed, such as the simple and reproducible high-performance thin-layer chromatographic (HPTLC) method of Puscas et al. [23], which is based on the physicochemical properties, δ^13^C signature, fructose/glucose ratio, and sucrose content evaluation [24,25].

Fingerprint techniques, such as chromatographic, spectroscopic (nuclear magnetic resonance (NMR), VIS, FTIR, Raman, NIR, and MIR), and electrochemical, are more practical for detecting honey adulteration, as they require little or no sample processing, use green reagents, and the data can be easily and more rapidly acquired compared to the quantitative methods [10,26]. Standalone, spectroscopic techniques are not always successful in identifying adulterant markers; thus, coupling them with chemometric tools is a more rational approach [27,28,29]. Reliable in pinpointing adulterants, NMR spectroscopy provides spectra in less than 5 min and offers simultaneous quantification of several components from a single run [18]. However, its broad-scale applicability is limited by its high acquisition and operating costs, need for skilled operators, and data processing requirements.

The MIR (4000–450 cm^−1^) region, with narrower and better-resolved fundamental vibrations, and the NIR (10,000–4000 cm^−1^) region, with information on high-frequency complex overtones and overlapping of vibrational peaks, have often captured hidden information on honey composition [30].

Gallardo-Velázquez et al. [31] used Fourier-transform infrared spectroscopy with attenuated total reflectance (ATR–FTIR) and the partial least squares (PLS) method to quantify CS, HFCS, and inverted sugar in Mexico honeys. Their soft independent modeling class analogy (SIMCA) models gave correct classification with zero false positive results. The 1300–1800 nm bands, characteristic for water and carbohydrates, include the most valuable information [32], allowing adulteration detection of acacia honey samples containing 10–90% sugar syrup. Spectral information was processed by principal component analysis (PCA), PLS, and artificial neural networks (ANNs) [33]. 

The IR region has been proven satisfactory in assessing the botanical origin and predicting the quality [34] or even the harvesting year of monofloral honey samples [35].

The NIR spectra of pure and adulterated (with 10–90% glucose syrup) acacia honey samples were analyzed [33]. Adulteration detection was based on NIR data processed using PCA, whereas PLS and ANN were employed to evaluate the adulteration level and changes in physicochemical characteristics (moisture content, conductivity, total color change, total phenolic content, and antioxidant activity measured by the FRAP).

Irradiation and aging of citrus, canola, macadamia, sunflower, and eucalyptus honey samples tainted with 10% and 20% HFCS and ICSS was found from their NIR spectra [36].

Broader investigation domains, such as UV–Vis (325–900 nm) and NIR (904–1699 nm), allowed the design of ANN models that were able to detect the adulteration of acacia honey with 10–90% CS, moisture, color, conductivity or antioxidant capacity, and total phenolic content [37].

There was a convincing demonstration that the UV–Vis region may offer valuable spectral windows, especially the 280–300 nm domain, for classifying mixtures of 139 Brazilian polyfloral honeys from different harvest seasons, containing 10–60% GS [38]. The authors advocated for the creation of a scalable national database dedicated to honey quality monitoring, with attention being given to the emerging adulterants.

Data pretreatment (autoscaling, mean-centering, first derivative, second derivative, multiplicative scattering correction, or adaptive iteratively reweighted penalized least squares—airPLS) was performed on the Raman [39], NIR [27], and UV–Vis [37] spectra to optimize the discriminant models before subjecting them to PCA and PLS–LDA.

The Raman data were also tested for botanical authentication in connection with LDA [40], as well as adulteration detection by 5–50% addition of FS, GS, IS, HFIS, and malt must [41].

Differential scanning calorimetry (DSC) data and sugars composition obtained using HPLC-RID offered the base for quantification of sunflower honey adulteration with 5–20% agave, maple, rice and corn syrups, or ISS [42], suggesting that DSC alone cannot offer sufficient information for correct classification. Same adulteration conditions were successfully identified for a set of four monofloral honeys, based on the information obtained using excitation–emission spectroscopy and PCA, PLS–DA, and support vector machines—SVMs [43].

This paper presents the results obtained in identifying the adulteration level of six Romanian honey types (five monofloral and one polyfloral) mixed with 5–50% CS, IS, and other commercially available syrups, such as corn syrup with plant extracts and fruit syrups, based on their UV–Vis spectra. Unsupervised and supervised classification instruments were used to select the information-carrying spectral features, which were further employed to develop a neural model that was able to identify and quantify adulteration levels.

A relatively large number of honey samples of different botanical origins, adulterated with commonly used commercial syrups, were investigated to prove the capability of UV–Vis spectral information when coupled with chemometric tools in providing information regarding the adulteration level. Several machine learning instruments were used for this purpose and compared in terms of their accuracy in identifying and quantifying honey adulteration.

## 2. Materials and Methods

### 2.1. Samples

Pure linden, acacia, rape, sunflower, honeydew, and polyfloral honeys (2018 harvest) were selected from the Vâlcea county. The samples were stored in airtight containers in a dark room, at room temperature. Prior to dissolution, the samples were checked for crystallization, mildly heated at 50 °C to remove any solid phase, and then centrifuged at 6000 rpm (1610× *g*) for 5 min.

Different syrups, including corn syrup, corn syrup with plant extracts, inverted syrup, and fruit syrup (Dulcofruct, Focsani, Romania) were used for direct adulteration of honeys with 5%, 10%, 20%, 30%, 40%, and 50% syrups (w:w). The adulterated honey samples were mechanically homogenized prior to spectral measurements.

### 2.2. Spectra Acquisition

The solvent used for dissolution, dilution, and baseline purposes was 80% ethanol in water (v:v) prepared from absolute ethanol with analytical grade quality (>99%) (Merck Co., Darmstadt, Germany) and ultra-pure water was produced by a Milli-Q Millipore system (Bedford, MA, USA). The 10% (w:v) stock solutions of samples were filtered through a 0.45 µm PTFE membrane, diluted 1:10 (v:v), and subjected to spectrophotometric measurements. Spectra were recorded on a UV–Vis spectrophotometer Specord 250 Plus (Analytik Jena, Jena, Germany), in 1 cm quartz cell, at 1 nm, against 80% aqueous ethanol solution as blank, in the 200–450 nm wavelength range. Temperature varied in the 23 ± 1 °C domain during records. Each sample was analyzed in triplicate. 

### 2.3. Data Processing

The UV–Vis spectra were recorded with the support of the WinASPECT PLUS software 221:335.28 Version 4.2.0.0 (Analytik Jena, Jena, Germany), exported to Microsoft Excel^®^ 365 (Microsoft, Redmond, WA, USA), averaged for each sample (*n* = 3), and further used for chemometric analysis.

Multivariate statistical analysis of the spectral data was conducted using principal component analysis (PCA), linear discriminant analysis (LDA) [44], partial least squares regression (PLS) [45], and neural modeling using in-house-developed procedures written in Matlab^®^ R2023b (MathWorks, Natick, MA, USA) software.

Since 250 datasets of spectra in the 200–450 nm range were to be analyzed, PCA was the instrument used for variable reduction, providing insight to unsupervised samples grouping.

LDA used predefined groups: pure honeys, syrups, and honeys adulterated with 5% up to 50% syrup. No differentiation was considered between the honey types or the syrup used, as the main goal of the classification was to find the degree of honey adulteration, regardless of the adulterant. LDA was performed using the first 20 principal components (PCs) for the samples’ characterization to avoid mathematical issues in matrices inversion.

PLS was applied to verify if a linear correlation could be established between the degree of adulteration and the UV–Vis spectrum using the investigated wavelength region. There were 84 samples (pure honey and adulterated ones) in the training set, while 7 samples were employed for testing. The input matrix X of independent variables was built using the UV–Vis spectral data of the training set in the 200–450 nm range. The dependent variables vector, Y, consisted of the corresponding adulteration levels. As the number of independent variables was larger than the number of samples, PLS, similar to PCA, calculated latent variables that maximized the covariance between X and Y vectors and used them to perform the linear regression. Over 95% of the experimental differentiation between samples’ characteristics was expressed in 18 latent variables. As the PLS algorithm subsequently distributes the values of correlation coefficients over the wavelengths characterizing the samples, the regression model can be directly applied for new samples, provided that their UV–Vis spectra are available. The quality of the regression model was appreciated by calculating the mean and maximum absolute error in the training and test sets. The error is expressed as an absolute difference between the experimental concentration of adulterant (%) in the sample and the value estimated by the regression model. The mean absolute error, err_mean_, was calculated using Equation (1).

(1) $${\mathrm{err}}_{\mathrm{mean}}=\frac{\sum_{i=1}^{N}\mathrm{abs}\left(y_{\exp,i}-y_{\mathrm{estimated},i}\right)}{N},$$

where y_exp_ is the sample adulteration degree (%) and y_estimated_ is the value delivered by the PLS regression model. N stands for the number of samples considered.

The identification of the variables (wavelengths in the UV–Vis spectra) with strong influence upon the adulteration degree was carried out by the “variable influence on projection (VIP)” calculation [46].

Further attempts for adulteration quantification were based on a neural network model of pattern recognition type built in the frame of Matlab 2023b software, using a Levenberg–Marquart algorithm for attaining convergence. The input vectors were represented by the first 20 PCs, encompassing the variability in the spectral data of 91 samples (pure honeys and adulterated ones). The output matrix contained 7 columns for the considered adulteration degree: 0% adulterant, pure honey—Class 1; 5% adulterant—Class 2; 10% adulterant—Class 3; 20% adulterant—Class 4; 30% adulterant—Class 5; 40% adulterant—Class 6; and 50% adulterant—Class 7. The network training was conducted using 70% of the dataset, testing used 15%, and validation used 15% to avoid overlearning. The network architecture giving the best results consisted of 20 input nodes (the 20 PCs scores), 12 neurons in the hidden layer, and 7 output nodes (the 7 classes). The training function was “trainscg” (scaled conjugate gradient backpropagation), implemented in Matlab R2023b software. Classification with the aid of such a network requires that each sample targets a class it should be attributed to. A 7-positions vector is defined, where the class position is 1 and the rest are 0. The vector for class 1 is (1, 0, 0, 0, 0, 0, 0), whereas that for class 7 is (0, 0, 0, 0, 0, 0, 1). 

## 3. Results and Discussion

The visual spectra inspection pinpoints the 200–400 nm region as a carrier of useful information. The 250–350 nm region of the UV–Vis absorption spectra is of interest, being associated with polyphenols and volatile compounds, originating from the honey floral sources [47] and propolis [48]. The strong absorption band around 280/290 nm observed in the UV–Vis spectra of sugar syrups can be associated with the colorless phenolic acids, flavanol monomers, polymers, etc. The studied honeys were previously characterized in terms of phytochemical composition [43], indicating that they contain significant amounts of phenolic acids (3,4-dihydroxybenzoic, *p*-hydroxybenzoic, caffeic, chlorogenic, syringic, *p*-coumaric, ferulic, and *t*-cinnamic acids) and flavonoids (quercetin, kaempferol, isorhamnetin, apigenin, pinocembrin, galangin, chrysin, and pinostrobin).

**Figure 1:**
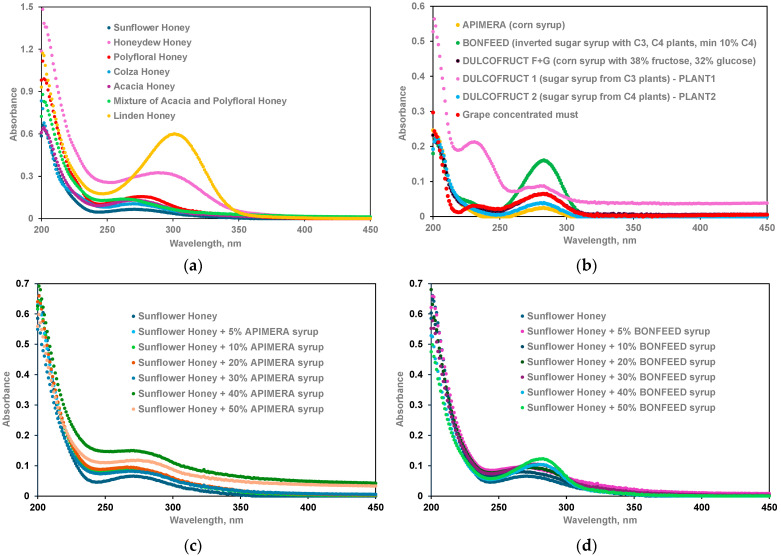
UV–Vis spectra of (**a**) honeys, (**b**) syrups, (**c**) sunflower honey adulterated with 5–50% corn sugar (APIMERA), and (**d**) sunflower honey adulterated with 5–50% enzymatic inverted sugar (BONFEED).

Adulteration of honeys with 5–50% corn syrup or enzymatic inverted sugar does not induce spectral changes directly correlated to the adulteration level (Figure 1).

The selected chemometric methods aimed to increase the differentiation between the recorded spectra. Adulteration identification was conducted using unsupervised and supervised approaches, such PCA and LDA. PLS regression and ANN models were used for adulterant quantification.

**Figure 2:**
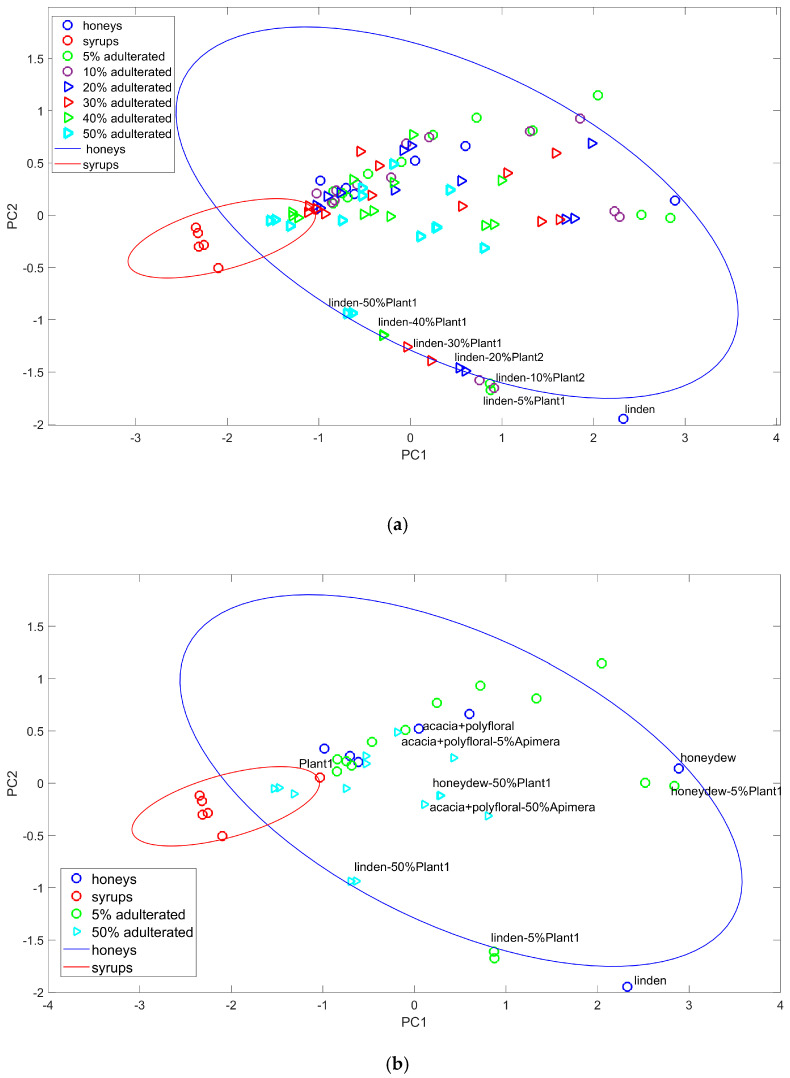
Honeys, syrups, and adulterated samples in the PC1–PC2 projection. (**a**) Overall picture of the investigated samples. (**b**) Close-up presenting the shift of samples with extreme levels of adulteration (5 and 50%) in the PC1–PC2 plane.

**Figure 3:**
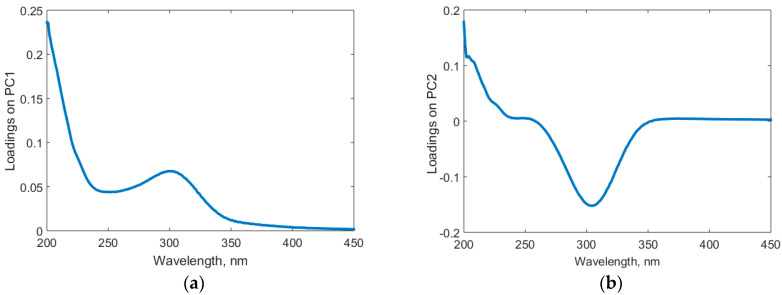
Wavelengths loadings on the principal components: (**a**) PC1, (**b**) PC2.

The spectra processed by PCA underwent significant variable reduction. The first two principal components encompassed more than 95% of the variability (PC1 74%, PC2 for 21.5%, Figure 2) with only 3% contribution from PC3. The input data representation in the PC1–PC2 coordinates (Figure 2a) shows a clear distinction between the syrups and the honey samples. However, the “Plant1” syrup sample (Figure 2b), along with pure honeys and adulterated ones, formed a large group. It becomes clear that the PC1–PC2 projection could not differentiate samples according to the adulteration level. Nevertheless, the new latent variables, PC1 and PC2, could differentiate pure honey of a given botanical type from its 5% and 50% adulterated variations (pure linden honey and adulterated samples marked in Figure 2a). Two other cases (honeydew and acacia–polyfloral mixture), as shown in Figure 2b, behaved similarly. This observation led to the hypothesis that a supervised classification tool could better differentiate the samples. As for the wavelength domains contributing to the samples’ differentiation, the 280–320 nm and 200–240 nm ranges proved to be more important due to their higher loads on PC1 and PC2 (Figure 3).

Frausto-Reyes et al. [49] reported that the first two PCs accounted for 99.5% of variability in the UV–Vis spectra of a series of monofloral and polyfloral honeys from Mexico, not considering adulterated mixtures. The first two PCs also encompassed 96% of the spectral variability (in the 260–360 nm region) of pure and 10–60% glucose-adulterated polyfloral honey samples from the Santa Caterina state, Brazil [40].

**Figure 4:**
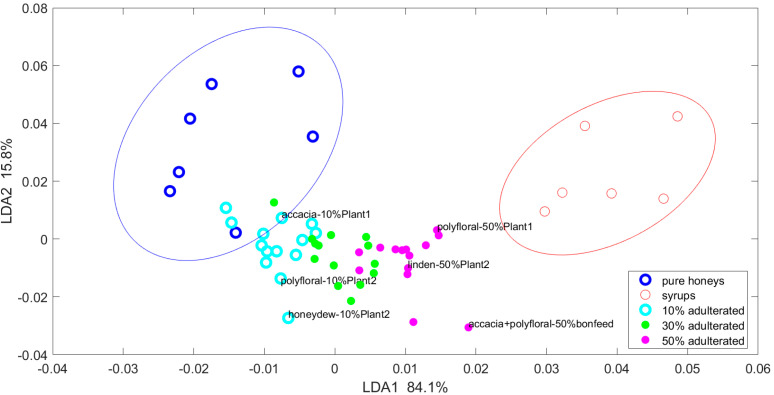
Samples differentiation in the first two coordinates identified by LDA.

The classification is much better evidenced by the LDA method. A number of 20 PCs, encompassing 99.99% of the experimental variability, were used as input variables in the LDA method to increase samples differentiation. The results shown in Figure 4, where the 10%, 30%, and 50% adulterated samples are represented, enable the visualization of distinct groups. The chosen degrees of adulteration can be considered as threshold limits for low-, medium-, and high-adulterated honeys.

As shown in Figure 4, the pure honeys were well separated from the adulterated ones, except for a single pure honey sample, which joined the group of 10% adulterated honeys. The medium-level adulterated honey samples were located between the low- and high-level adulterated ones, with very little overlapping with adjacent groups. All syrups were completely differentiated from the honey samples. The differentiation on the first LDA function, which encompassed 84% of data variability, was mainly noticed for the different adulteration degrees. The differentiation according to the botanical origin of the adulterated honey samples was less prominent, being mainly noticed along the second LDA function, which encompassed 15.8% of the variability. This observation demonstrates that UV–Vis spectra provide information mainly related to the differences between the adulteration degrees of pure honeys, irrespective of their botanic origin, and thereby confirm their possible utility in honey adulteration control. This is in line with the findings of Valinger [31] and Nunes [38].

### 3.1. Partial Least Squares Regression (PLS)

**Figure 5:**
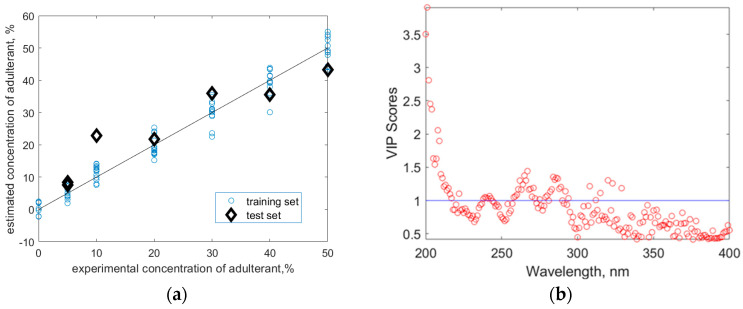
(**a**) Model parity plot obtained using PLS regression. (**b**) VIP scores for the tested spectral range.

The linear correlation between the adulteration degree and the UV–Vis information delivered by PLS was accompanied by a 2.64% and 5.4% mean absolute error in training and testing, respectively. The maximum error in training was 9.8% of adulterant identified, whereas that in testing was 12.9% of adulterant (experimental value 10%, estimated value 22.9%). The graphical representation of the accuracy of the PLS regression model is presented in the parity plot diagram (Figure 5a). Calculation of VIP scores showed that the most important wavelengths in defining the adulteration degree are in the 200–320 nm range (Figure 5b), where VIP scores are greater than 1. This is consistent with the first two PCs loadings shown in Figure 3. As shown in Figure 5b, the contribution of wavelengths higher than 320 nm is also significant, as several VIP scores exceeded 0.5, with some being rather close to 1. Therefore, the prediction is improved if all the spectral information is used.

### 3.2. Artificial Neural Networks (ANNs)

Classification by ANNs considered seven classes: honey—class 1; honey adulterated with 5% syrup—class 2; honey adulterated with 10% syrup—class 3; honey adulterated with 20% syrup—class 4; honey adulterated with 30% syrup—class 5; honey adulterated with 40% syrup—class 6; and honey adulterated with 50% syrup—class 7. The first 20 PCs of the samples from these classes were used for building the neural model. 

**Figure 6:**
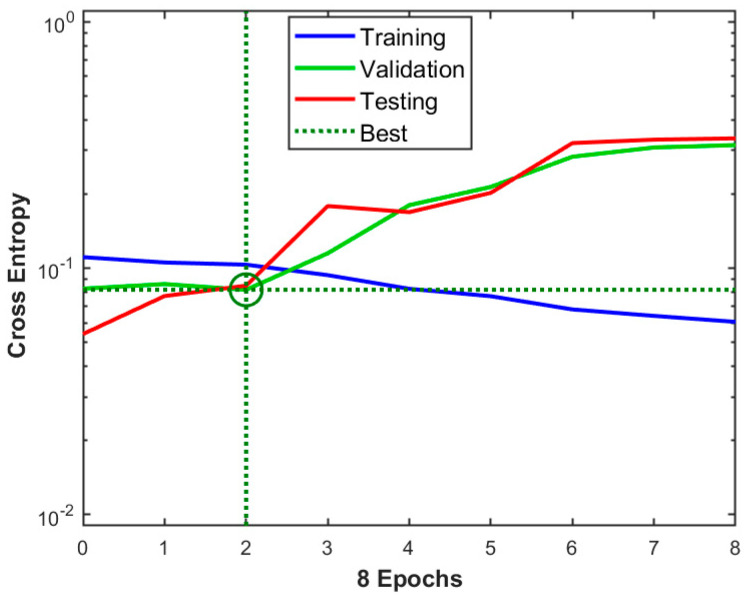
ANN performance for the proposed model.

The results obtained during the network build-up can be visualized by representing the performance evolution during training, validation, and testing (Figure 6). The “Cross Entropy” represents the overall capability of the model to capture the data feature. Its best value, 0.081625, corresponds to epoch 2, where there is a trade-off between the training, validation, and testing performance.

**Figure 7:**
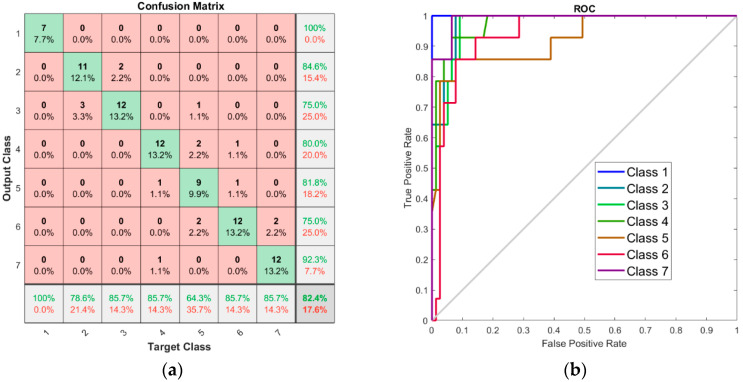
(**a**) Confusion matrix for the ANN model. (**b**) ROC curve for the ANN model.

The performance of the ANN model in pattern recognition can be seen from the confusion matrix (Figure 7a) and receiver operator characteristic (ROC) curve (Figure 7b). The latter represents the variation of the true positive cases (sensitivity) versus the false positive ones (specificity). As shown in Figure 7a, pure honey samples (class 1) were correctly assigned, and no adulterated sample, even those with only 5% adulterant, was “seen” as pure honey.

The accuracy (shown in the last cell of the confusion matrix) of adulteration quantification is 82.4%. The true positive rate (recall), calculated on the last line of the matrix (in green), was the lowest for class 5. This means that a larger number of 30% adulterated honeys were not attributed to the corresponding class (from 14 honey samples adulterated with 30% syrup, only 9 were identified as such by the classification model). The accuracy, calculated in the last column (in green), is lowest for class 6, indicating that from all samples “seen” as being 40% adulterated honeys by the classification model, a relatively smaller number had that actual adulterant concentration (i.e., although 16 samples were attributed to class 6, only 12 were really 40% adulterated).

It must be noted that almost all misinterpretations of the adulteration level occurred due to assigning the investigated sample to a class adjacent to its real one. There are few exceptions, though: one sample experimentally adulterated with 20% (class 4) was attributed to class 7 (50% adulteration), another sample from class 5 (30% adulteration) was assigned to class 3 (10% adulteration), and a sample from class 6 (40% adulteration) was assigned to class 4 (20% adulteration).

More insight to appreciate the classification model can be found by representing the adapted ROC curves and evaluating the area under the curve (AUC), which should be as close to 1 as possible. This technique is generally defined for binary classification models; however, it may be adapted for multiclass models as well. For a multiclass model, the OvR (one versus rest) ROC curves were plotted considering each class compared to the rest. These curves were plotted for several thresholds between 0 and 1, while considering the identification of an analyzed sample in the correct class as a positive answer, and its attribution to any other class as a negative one.

The area under the ROC curve for class 5 (30% adulteration) in Figure 7b is the lowest, followed by the surface described by class 6 (40% adulteration). This means that the ANN classification model obtained is less capable for the exact quantification of a sample with 30% adulteration than those with very low (10%) or high (50%) adulteration. This result is in good agreement with the samples classification by LDA, where the 30% adulterated samples overlapped the 10% and 50% adulterated honeys.

These observations prove that the ANN model has good accuracy in identification of adulterated honeys, with a reasonable adequacy for the quantification of the adulterant.

Newly prepared adulterated honeys were scanned in the UV–Vis region for a supplementary validation of the ANN model. The spectral data in the 200–450 nm range were projected on the principal components obtained in the frame of PCA, and the first 20 PCs scores were used as input values in the ANN model. The classification of these supplementary samples is presented in Table 1.

It can be seen from Table 1 that 4 samples out of 17 were incorrectly assigned to adjacent classes (marked in bold). This supplementary test proved that the ANN model can identify the adulteration of honey, and the error in the adulteration degree estimation may be approximately 10%.

Dimakopoulou-Papazoglou et al. [50] also applied PCA followed by a random forest (RF) algorithm, partial least squares–discriminant analysis (PLS–DA), and data-driven–soft independent modeling of class analogies (DD-SIMCA) to predict the adulteration of mediterranean honeys based on selected UV wavelengths. Their higher 92% accuracy was achieved using exclusively thyme Greek honey in the validation set.

The different chemometric methods used in the present study proved that UV–Vis spectral information can be a reliable base for honey adulteration identification and quantification. Applying these methods to spectral data obtained for honeys of different botanical origins and six different adulterant syrups proves the applicability of this approach in practical applications aiming to test various commercial honey samples.

As UV–Vis spectral data represent too large a matrix to be directly used in supervised methods, such as LDA and neural modeling, data reduction proved to be necessary. The unsupervised machine learning tool, PCA, was successfully used for data reduction. The new uncorrelated variables obtained were used when LDA and a neural model were applied. New samples require variables reduction by projection in the PCA latent variables. These new PCs can be further projected onto the LDA functions to visualize the test samples class membership, or loaded as input data into the neural model to calculate the assigned adulteration class. The proposed PLS regression model has the advantage of being easy to use for quantifying the adulteration level of new samples based on their UV–Vis spectral data. The performance of the presented machine learning techniques will certainly improve once a larger spectral database becomes available, which will include not only the botanical, but also the geographical, seasonal, and climatological, variability of the targeted honey samples and the syrups involved in the apicultural practices.

## 4. Conclusions

UV–Vis spectra, a rapid, simple, and cost-efficient analysis tool, were successfully used for the evaluation of honey quality, in terms of possible adulteration, by means of using adequate chemometric tools and classification models. The variety of honeys and syrups used to design the mixtures subjected to the chemometric analysis advocates the possibility of successfully applying the proposed procedures in the verification of any commercial honey sample. Various machine learning algorithms were tested—PCA–LDA, PLS, and neural modeling—and the results obtained confirmed their capability in identifying different adulteration levels.

More than one processing routine was used to obtain cross-validation and enhancement of results. The selection of tools was conducted in connection with the input data and the aim of the investigation. 

Comparing the results obtained by the different machine learning tools, the present study evidently proves that an unsupervised method, such as PCA, when applied to UV–Vis spectral data, cannot provide a sample separation according to the adulteration degree. Only large differences between pure and adulterated honeys on one side and syrups on the other side can be evidenced in the PC1–PC2 space. PCA was just a useful tool for data reduction. The supervised classification method, LDA, could separate only samples with significant differences in adulterant concentration (~20%). LDA proved that classes corresponding to an adulteration level within this range can be identified irrespective of botanical origin of honey and syrup type.

The PLS method applied to UV–Vis spectral data ensured an overall mean error of approximately 10% adulterant concentration.

A more elaborate classification model based on ANN structure gave the best results in identifying the degree of honey adulteration. These models also require spectral data preprocessing to obtain a reasonable number of inputs necessary for training the network. The classification ANN model was based on the variability encompassed in the first 20 PCs. The ROC curve for the ANN model revealed that samples with low- and high-adulteration degrees can be correctly classified, with only a small number of false positives and false negatives. The intermediate adulteration levels have a higher chance to be attributed to adjacent classes. The ANN classification model was found to be the most adequate tool for adulteration quantification, and, by increasing the datasets, the accuracy of the results will also increase.

The proposed models were able to identify mixtures of pure honey and sugar syrups with detection limits higher than 10%. Even though the precision of adulterant quantification is not very high, the easiness of spectral investigation makes it attractive for rapid honey investigation.

## Figures and Tables

**Table 1:** Experimental and attributed classes by the ANN model for supplementary adulterated honey samples.

Class 2		Class 4		Class 7	
Experimental	Attributed	Experimental	Attributed	Experimental	Attributed
2	2	4	4	7	7
2	2	4	4	7	**6**
2	**3**	5	**6**	7	7
3	3	5	5		
3	3	6	6		
3	3	6	6		
4	4	6	**7**		

## Data Availability

The original contributions presented in the study are included in the article, further inquiries can be directed to the corresponding author.
